# Efficacy of total arch replacement with frozen elephant trunk in patients with acute type A aortic dissection

**DOI:** 10.1016/j.xjon.2025.07.002

**Published:** 2025-07-16

**Authors:** Yosuke Inoue, Kazufumi Yoshida, Yojiro Koda, Takayuki Shijo, Yoshimasa Seike, Hitoshi Matsuda

**Affiliations:** Department of Vascular Surgery, National Cerebral and Cardiovascular Center, Suita, Japan

**Keywords:** total arch replacement, frozen elephant trunk, aortic dissection, mid-term

## Abstract

**Objective:**

To compare the outcomes of classical elephant trunk (CET) + total arch replacement (TAR) and frozen elephant trunk (FET) + TAR using propensity score matching analysis.

**Methods:**

Between 2012 and 2023, 370 patients who underwent TAR were divided into 2 groups based on their elephant trunk type: the CET group (153 patients; 92 men; mean age, 66 ± 13 years) and FET group (217 patients; 116 men; mean age, 64 ± 12 years). Among these patients, 124 from each group were matched using propensity scores to account for differences in patient characteristics.

**Results:**

Early outcomes, such as mortality and morbidity, were similar between the unmatched and matched cohorts. Circulatory arrest time was significantly shorter in the CET group, even after propensity score matching. In matched cohorts, the FET group had significantly higher rates of freedom from dissection-related distal aortic reoperation at 3 years and 5 years (87% and 85%, respectively, in the CET group and 96% and 96%, respectively, in the FET group; *P* = .008). Cox regression analysis identified the FET procedure (hazard ratio, 0.20; *P* = .008) is an independent positive inhibitory factor of distal aortic reoperation. Serial sizing analysis revealed that the aortic diameter at the level of the celiac artery was significantly smaller in the FET group even 5 years after the initial surgery.

**Conclusions:**

FET + TAR has potential as the first option for improved mid-term outcomes after surgery for type A acute aortic dissection.

**Figure:**
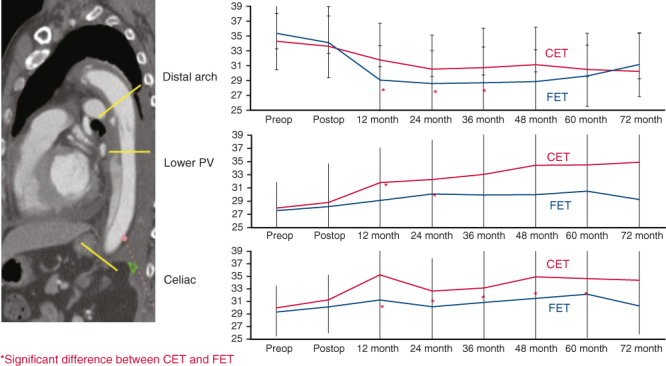
Differences in aortic diameter after CET versus FET.

**Central Message**

Frozen elephant trunk procedures promote false lumen thrombosis of the thoracic aorta, leading to the prevention of aortic enlargement even at the level of celiac artery and distal aortic reoperation.

**Perspective**

The total arch replacement (TAR) classical elephant trunk technique has the potential to become a standard procedure with guaranteed long-term results if the acute mortality rate can be reduced to a comparable level to that seen in non-TAR procedures with further evolution and improvement of the device.

Total arch replacement (TAR) with frozen elephant trunk (FET) (TAR + FET) is a viable approach for managing patients with type A acute aortic dissection (AAAD), promoting false lumen (FL) thrombosis at the distal aorta and eventually leading to aortic remodeling.^1^^,^^2^ However, owing to concerns about postoperative spinal cord ischemia (SCI) and the formation of distal stent graft induced new entry (d-SINE) during follow-up periods, FET has not been recommended in the guidelines.^3^ We previously published data on the early outcomes of TAR + FET compared to the conventional TAR procedure using classical elephant trunk (CET; TAR + CET),^4^ but the mid-term results of TAR + FET are unknown. This study was conducted to compare the mid-term results of TAR + FET and TAR + CET using propensity score matching (PSM) analysis.

## Materials and Methods

### Ethics Statement

This retrospective observational study was authorized by the National Cerebral and Cardiovascular Center's Institutional Review Board (M30-057; approved September 5, 2018). The need for patient informed consent was waived owing to the study's retrospective nature.

### Patients

Between January 2012 and December 2023, a total of 675 patients underwent emergency AAAD surgery within 14 days of onset at our facility. Among them, 370 patients who underwent TAR were enrolled, after 305 patients were excluded for the following reasons: thoracic endovascular repair (TEVAR) in 22 patients, and a non-TAR procedure, including isolated hemiarch replacement and partial arch replacement with reconstruction of 1 artery (brachiocephalic) or 2 arteries (brachiocephalic and left carotid) in 283 patients. Patients with DeBakey type II (dissection located only in the ascending aorta) were excluded from this study and underwent non-TAR procedures. Finally, patients were divided into 2 groups based on basic surgical strategy: TAR + CET group (n = 153) and TAR + FET group (n = 217).

**Figure 1:**
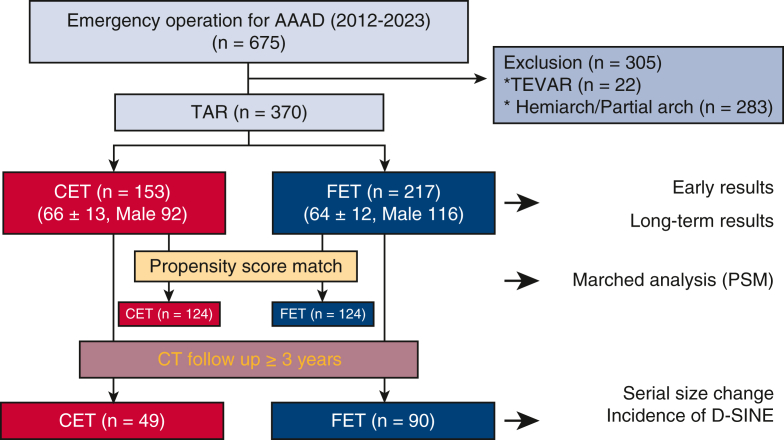
Flow chart of study inclusion. After excluding patients based on the listed exclusion criteria, 370 patients were ultimately enrolled in the study. *AAAD*, Acute type A aortic dissection; *PSM*, propensity score matching; *CET*, classical elephant trunk; *FET*, frozen elephant trunk.

Among the 370 patients, PSM used 8 preoperative variables to match each of the 124 patients. Furthermore, serial aortic diameters at each segment of the downstream aorta were compared in patients followed up for longer than 3 years after the initial operation (Figure 1).

In the unmatched cohort, the TAR + FET group had significantly more patients with organ malperfusion compared to the TAR + CET (*P* = .002). Furthermore, the TAR + FET group experienced more severe distal aortic dissection (*P* < .001). Table 1 shows patient characteristics for both the overall and propensity score–matched cohorts.

**Table 1:** Preoperative patient characteristics

Characteristic	Overall				Matched			
	CET (N = 153)	FET (N = 217)	*P* value	SMD	CET (N = 124)	FET (N = 124)	*P* value	SMD
Age, y, mean ± SD	66 ± 13	64 ± 12	.91	0.093	64 ± 13	65 ± 12	.60	0.066
Males: females, n	92:61	116:101	.24	0.135	75:49	68:56	.44	0.114
Preoperative status, n (%)								
Shock	32 (21)	24 (15)	.16	0.015	22 (17)	20 (16)	.87	0.041
CPR	7 (4.5)	9 (4.1)	1	0.15	5 (4.0)	6 (4.8)	.77	0.040
Organ malperfusion, n (%)	53 (35)	113 (52)	.002	0.35				
Coronary	10 (6.5)	22 (10)	.26	0.13	10 (35)	9 (52)	1	0.030
Brain	27 (18)	61 (28)	.025	0.25	23 (6.5)	26 (28)	.75	0.061
Visceral	9 (5.8)	28 (13)	.034	0.24	9 (5.8)	11 (13)	.81	0.059
Connective tissue disorder, n (%)	14 (9.3)	12 (5.6)	.24	0.14	11 (9.0)	5 (4.1)	.19	0.020
Serum creatine ≥1.5, n (%)	7 (4.7)	16 (7.8)	.24	0.14	5 (4.1)	12 (9.9)	.13	0.23
Dissection classification, n (%)								
DeBakey type I	114 (76)	152 (70)	.34	0.13	97 (78)	92 (74)	.46	0.132
DeBakey type IIIbR	25 (16)	51 (23)	.12	0.17	24 (20)	35 (28)	.14	0.20
Patent false lumen status, n (%)								
Descending aorta	103 (83)	172 (83)	.98	0.02	100 (84)	92 (77)	.25	0.171
Abdominal aorta	83 (88)	155 (85)	.53	0.11	82 (88)	86 (85)	.68	0.089
Distal extent of dissection, n (%)								
Distal arch	32 (21)	5 (4)	<.001	0.63	5 (4)	5 (4)	.68	0.193
Descending aorta	19 (12)	19 (9)			17 (14)	15 (12)		
Abdominal aorta	24 (16)	36 (25)			24 (19)	31 (25)		
Iliac or beyond iliac	78 (51)	99 (62)			78 (63)	73 (59)		

*CET*, Classical elephant trunk; *FET*, frozen elephant trunk; *SMD*, standardized mean difference; *SD*, standard deviation; *CPR*, cardiopulmonary resuscitation.

### Indication of TAR and Patients’ Preparation

As reported previously, we have aggressively advocated for TAR beyond the tear-oriented approach for younger patients (age <50 years), patients with arch dilatation (>50 mm), and those with strong suspicion of connective tissue disease even with the primary entry located inside the ascending aorta.^5^ Preoperative spinal fluid drainage was not performed in any patient. All surgical maneuvers were performed via a median sternotomy according to previously reported methods.^5^ Double-arterial cannulation was routinely performed, primarily through the distal right axillary and left common femoral arteries.^6^^,^^7^ In patients with dissections that extended up to the right axillary artery or those who required cardiopulmonary resuscitation, a single femoral artery cannulation was used to establish cardiopulmonary bypass.

### Choice of Elephant Trunk and Principal Application Criteria for FET

In 2016, 2 years after introduction of the commercially available FET device (FROZENIX; Japan Lifeline Co, Ltd) was introduced in Japan, FET became the first choice for patients requiring TAR at our institution. The FET approach has been indicated primarily for patients who meet the following criteria: (1) patent FL at the descending aorta; (2) no evidence of connective tissue disease, such as typical physical characteristics, a family history of aortic dissection, and/or a previous definitive genetic diagnosis; and (3) primary entry found proximal to the eighth intercostal artery in patients with retrograde type A dissection. Those who did not meet the criteria underwent the same TAR + CET as was performed prior to 2016 (Video 1).

The following intraoperative principles were followed in patients who met the criteria for FET use: (1) FET was deployed during transient retrograde perfusion via a femoral artery cannula to flush out debris and air; (2) the FET size was selected based on intraoperative measurement of the aortic diameter just distal to the left subclavian artery, using a ball sizer to choose a diameter equal to or 1 mm larger than the measured size; (3) the FET was positioned just distal to the left subclavian artery (zone 3) and secured with continuous suturing reinforced by a felt strip, then anastomosed to the proximal prosthetic graft; and (4) a 9-cm-long stented portion of the FET was typically used.^8^ FROZENIX is separate type open stent graft with lengths of 6, 9, 12, and 15 cm.

### Definitions and Follow-up Data Collection

Data were gathered from hospital admissions and outpatient medical records. All patients with normal renal function underwent contrast-enhanced CT angiography (CE-CTA) within 1 month of surgery. Follow-up noncontrast CT scans were typically performed at the outpatient clinic or in several nearby hospitals at 3 to 6 months and 12 months after surgery and then annually thereafter. During the follow-up, further CE-CTA examination was considered in patients with significant aortic dilatation and/or morphologic changes around the distal portion of the elephant trunk. d-SINE is defined as a newly developed entry during the follow-up period that was not visible on early postoperative CE-CTA and cannot be ruled out as related to the elephant trunk.

Dissection-related distal aortic reoperation was described as reoperation due to d-SINE, aortic enlargement (rapid expansion; ≥5 mm within 6 months and/or distal aortic diameter ≥55 mm), or rupture of the descending and/or thoracoabdominal aortas. Reoperation for graft infection or isolated infrarenal abdominal aortic aneurysm repair was not considered dissection-related.

### Statistical Analysis

All statistical analyses were conducted with EZR (Saitama Medical Center, Jichi Medical University), a graphical user interface for R (R Foundation for Statistical Computing).^9^ Categorical variables were evaluated using the Fisher exact test, and continuous variables were analyzed using the *t* test for normal distribution (mean with standard deviation) or the Mann-Whitney *U* test for nonparametric data (median with range). Statistical significance was determined at *P* < .05 in the initial analysis. *P* values obtained were adjusted (*P*c) for multiple tests using Bonferroni correction for the number of tests. All-cause mortality and freedom from dissection-related distal aortic reoperation rates were calculated using the Kaplan-Meier method and compared using the log-rank test. To rule out the possibility of the perioperative course influencing midterm survival, a landmark analysis beginning 30 days after surgery was performed. A Cox proportional hazards model was used in multivariable analyses to assess the time-to-event effects of the covariates.

## Results

### Early Results (Entire Cohort)

The overall in-hospital mortality rate was 8.6% (n = 32/370), with no significant difference observed between the TAR + CET and TAR + FET groups (8.4% [n = 13/153] vs 8.7% n = 19/217]; *P* = 1.0). Circulatory arrest time was significantly shorter in the TAR + FET group (mean, 56 ± 12 minutes vs 65 ± 25 minutes; *P* < .001). There was no significant difference in the rate of stroke (12% for TAR + CET vs 17% for TAR + FET; *P* = .16) or renal failure requiring transient hemodialysis (2% vs 7.7%; *P* = .11). There were no cases of permanent SCI in either group, but 2 TAR + FET patients experienced transient SCI and were able to walk unassisted at the time of discharge. The postoperative results are shown in Table 2.

**Table 2:** Intraoperative variables and postoperative results

Variable	Overall			Matched		
	CET (N = 153)	FET (N = 217)	*P* value	CET (N = 124)	FET (N = 124)	*P* value
Operative parameters, min, mean ± SD						
Operation time	489 ± 165	484 ± 133	.79	496 ± 173	482 ± 137	.47
CPB time	282 ± 97	272 ± 87	.31	287 ± 100	268 ± 87	.11
Cardiac ischemic time	158 ± 58	164 ± 57	.31	158 ± 55	157 ± 60	.95
Circulatory arrest time	65 ± 25	56 ± 12	<.001	67 ± 26	57 ± 11	<.001
Lowest temperature, °C, mean ± SD	23.4 ± 2.0	23.7 ± 1.5	.19	23.3 ± 2.1	23.6 ± 1.5	.28
Primary entry resection				75:49	68:56	.44
Early results						
In-hospital mortality, n (%)	13 (8.5)	19 (8.8)	1	9 (7.3)	10 (8.1)	1
TND, n (%)	21 (14)	37 (17)	.47	18 (15)	19 (15)	1
PND preoperative + postoperative, n (%)	18 (12)	37 (17)	.21	14 (12)	21 (17)	.31
Disabling stroke (mRS ≥3), n (%)	6 (2.7)	4 (1.8)	.22	3 (2.4)	3 (2.4)	1
Spinal cord ischemia, n (%)						
Transient	0.2	2	.23	0	2	.35
Permanent	0.0	0	1	0	0	1
Temporary RRT, n (%)	18 (15)	15 (7.1)	.15	14 (12)	12 (9.7)	.77
Mechanical ventilation ≥72 h, n (%)	55 (93)	93 (43)	.20	43 (35)	48 (39)	.67
Tracheostomy	12 (25)	25 (12)	.32	9 (7.4)	14 (11)	.40
ICU stay, d, median [IQR]	6 [4-8]	7 [4-11]	.02	5 [4-7]	7 [4-10]	.047
Hospital stay, d, median [range]	30 [23-41]	29 [23-40]	.9	30 [23-41]	30 [24-40]	.85

*CET*, Classical elephant trunk; *FET*, frozen elephant trunk; *CPB*, cardiopulmonary bypass; *SD*, standard deviation; *TND*, transient neurologic deficit; *PND*, permanent neurologic deficit; *mRS*, modified Rankin Scale; *RRT*, renal replacement therapy; *ICU*, intensive care unit; *IQR*, interquartile range.

**Figure 2:**
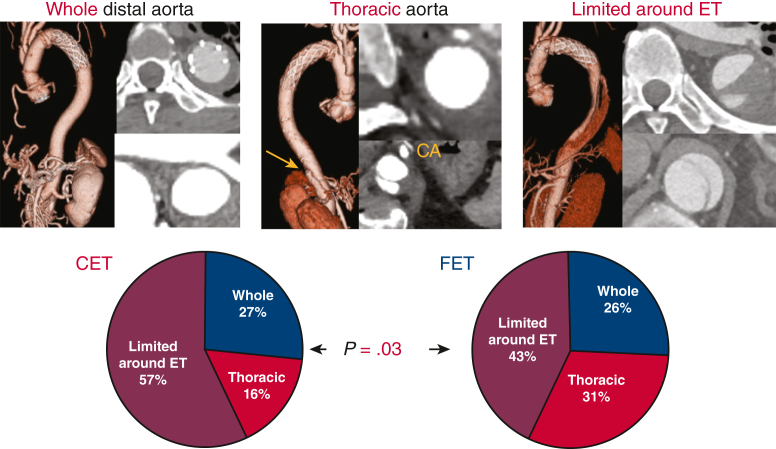
Pattern of false lumen thrombosis at downstream aorta in the 2 study groups. Frozen elephant trunk (FET) promoted false lumen thromobosis at total and thoracic aorta more in 57% than patients with classical elephant trunk (CET).

Figure 2 depicts the status of thrombosis inside the FL as detected by CE-CTA in the early phase at 1 month after surgery. FL Thrombosis of the entire thoracic and abdominal aorta occurred in 26% of the patients in each group. FL thrombosis in the thoracic aorta was significantly higher (31% vs 16%) and limited FL thrombosis around ET was significantly lower (43% vs 56%) in the TAR + FET group (*P* = .03).

### Late Results (Entire Cohort)

**Figure 3:**
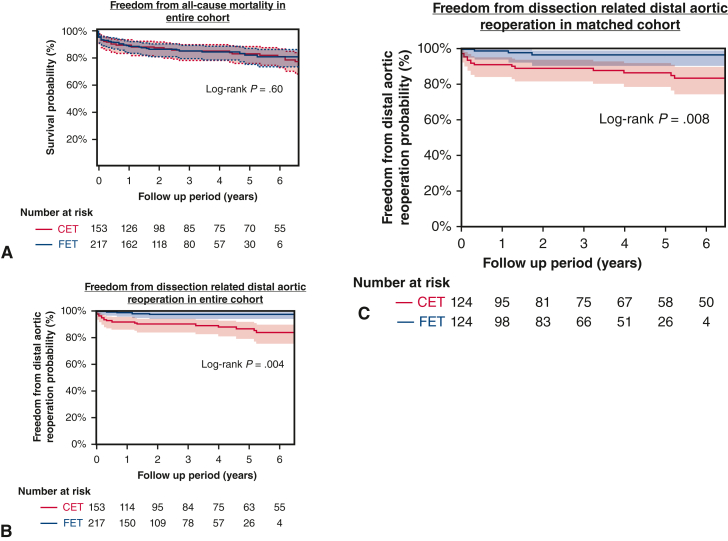
A, Probability of freedom from all-cause mortality in entire cohort (95% confidence interval [CI]), showing no significant difference between the classical elephant trunk (CET; *red*) and frozen elephant trunk (FET; *blue*) groups. B, Probability of freedom from dissection-related distal aortic reoperation in entire cohort (95% CI). The FET group (*blue*) showed a statistically higher rate compared to the CET group (*red*). C, Probability of freedom from dissection-related distal aortic reoperation in the propensity score–matched cohort with 95% CI, showing a statistically higher rate in the FET group (*blue*) compared to the CET group (*red*).

The TAR + CET group had a significantly longer mean follow-up compared to the TAR + FET group (62 ± 42 months vs 42 ± 27 months; *P* < .001). The freedom from all-cause mortality, including early death, was similar in the 2 groups (TAR + CET: 3 years, 85%; 5 years, 83%; TAR + FET: 3 years, 85%; 5 years, 82%; *P* = .79) (Figure 3, *A*). Twenty-one reoperations (9 replacements via thoracotomy, 12 TEVARs) were performed in the TAR + CET group, compared with 5 reoperations (2 replacements, 3 TEVARs) in the TAR + FET group. TEVAR was recommended for d-SINE treatment for only 1 patient in the TAR + FET group. Freedom from dissection-related distal aortic reoperation was significantly lower in the TAR + CET group (3 years, 88%; 5 years, 85%) compared to TAR + FET (98% at both time points) (*P* < .001) (Figure 3, *B*).

### Analysis of Matched Cohorts by Propensity Score Matching

There were no significant differences in preoperative patient characteristics among the 124 propensity score–matched pairs (Table 1). After matching, a balance check revealed comparable cofounders in the 2 groups (standardized mean difference <0.20). Early results, including in-hospital mortality and complications, were comparable in the 2 groups, except for significantly shorter lower body circulatory arrest time in the TAR + FET (mean, 57 ± 11 minutes; *P* < .001) (Table 2). The rate of avoidance of dissection-related distal aortic reoperation was significantly higher in the TAR + CET group (3 years, 87%; 5 years, 85%) compared to the TAR + FET group (96% at both time points; *P* = .008) (Figure 3, *C*). Cox regression analysis identified a residual primary entry tear (HR, 4.3: *P* = .07) and a postoperative patent FL in the descending aorta (HR, 2.59: *P* = .003) as risk factors for distal aortic reoperation following a dissection and revealed TAR + FET (HR, 0.20: *P* = .006) as a statistically significant inhibitor of dissection-related distal aortic reoperation (Table 3).

**Table 3:** Risk factors for dissection-related distal aortic reoperation

Variable	Univariable, *P* value	Multivariable	
		*P* value	HR (95% CI)
Age < 75 y	.14	.46	2.18 (0.27-17.7)
Remaining primary entry tear after operation	.03	.07	4.34 (1.61-11.1)
FET procedure	.008	.006	0.20 (0.06-0.64)
Connective tissue disease	.65	.46	0.47 (0.06-3.59)
Postoperative patent FL at descending aorta	.03	.003	2.59 (0.92-7.28)
Chronic kidney disease	.39	.09	3.81 (0.82-17.7)

*HR*, Hazard ratio; *CI*, confidence interval; *FET*, frozen elephant trunk; *FL*, false lumen.

### Serial Sizing Analysis

**Figure 4:**
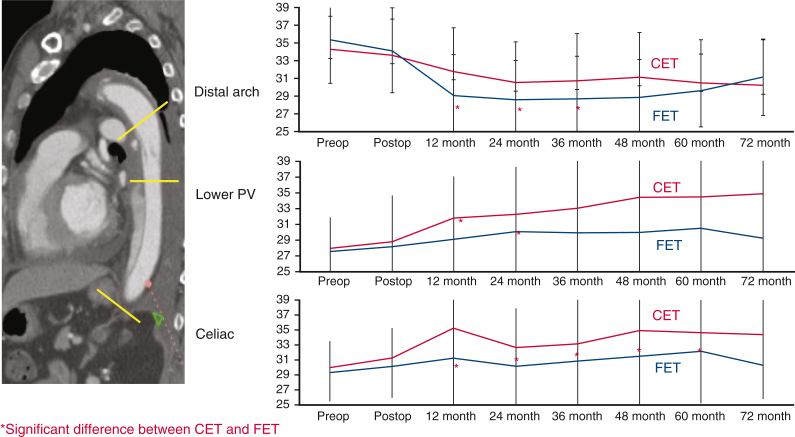
A, Serial size change of postoperative distal aortic diameter at each segment of aorta between the classical elephant trunk (CET) group (*red*) and the frozen elephant trunk (FET) group (*blue*).

**Figure E1:**
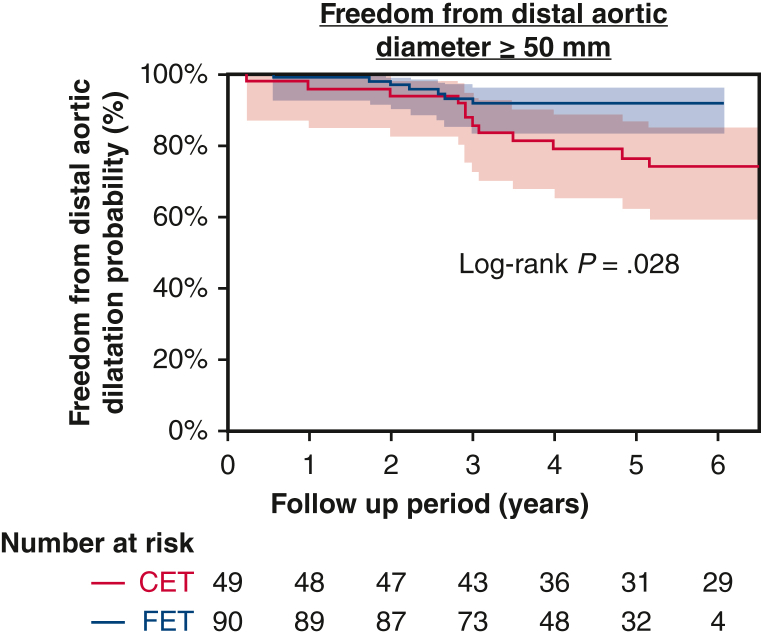
Probability of freedom from distal aortic diameter ≥50 mm (95% confidence interval) was statistically higher in the frozen elephant trunk (FET) group (*blue*) compared to the classical elephant trunk (CET) group (*red*).

A total of 139 patients (TAR + CET, n = 49; TAR + FET, n = 90) were monitored by CT scans for longer than 3 years after surgery. Serial changes in aortic diameter were measured at the tracheal bifurcation, left pulmonary vein, and celiac artery (Figure 4). In the TAR + FET group, aortic diameters tended to shrink significantly by 2 years after surgery at the distal arch and left pulmonary vein and for up to 5 years at the celiac artery level. The TAR + FET group had a significantly higher rate of freedom from a maximum aortic diameter ≥50 mm at 5 years (91% vs 72%; *P* = .028) (Figure E1). d-SINE occurred in 3 patients of the TAR + FET group, 1 of whom required additional TEVAR to treat rapid enlargement.

## Discussion

In AAAD surgery, hemiarch (or ascending aortic) replacement could resect primary entry tears in 70% of patients using the tear-oriented approach recommended by the EACTS/STS guidelines.^10^, ^11^, ^12^, ^13^ FET, which requires TAR, is currently recommended only for acute dissections that are non-A, non-B, or retrograde type A and have the primary entry in the aortic arch or descending aorta.

The postoperative patency of FL in the downstream aorta has been identified as a significant risk factor for distal aortic reoperation.^5^^,^^14^ If FET promoted FL thrombosis and aortic remodeling, then the increased invasiveness of TAR during initial surgery for AAAD would be justified by better long-term outcomes under conditions of comparable acute morbidity and mortality as seen with ascending aorta replacement. Indeed, benefits of TAR + FET for long-term outcomes have been reported in recent years.^15^ In contrast, it is reasonable to give priority to conventional TAR that does not use FET—that is, TAR + CET—because of the specific complications with FET, such as SCI and d-SINE. Comparing the 2 approaches is very important in terms of whether FET can be the first choice; however, no comparative study reporting the mid-term results of TAR + FET with background adjustment using PMS has been published to date.

The use of FET also allows for proximalization of the distal anastomosis of TAR. To enhance this benefit, surgical techniques vary, as do the indications for FET, which differ depending on the location and thrombosis of the FL, hindering comparisons between TAR + CET and TAR + FET. In this study, based on consistent surgical procedures—including zone 3 distal anastomosis in both groups and device diameter and length—perioperative patients' backgrounds differed, including the proportion of organ malperfusion and extent of aortic dissection. To compare the 2 techniques as fairly as possible, PSM was performed to validate the value of FET in mid-term outcomes by adjusting the patients’ backgrounds.

Lower body circulatory arrest time is one of the significant differences between the 2 study groups. Compared to the TAR + CET procedure, which routinely requires graft–graft anastomosis owing to a stepwise technique, the TAR + FET procedure itself is hemostatic procedure without additional graft–graft anastomoses that might contribute to reduced circulatory arrest time.

SCI is a major concern with TAR + FET, and several risk factors have been identified, including air embolism during FET deployment and low perfusion of the intercostal artery caused by an FL.^3^^,^^16^^,^^17^ Several reports have identified FET use as a risk factor for SCI in the entire cohort but not in a cohort limited to AAAD with a length <15 cm.^18^^,^^19^ In the current AAAD study, permanent SCI was not observed in either group. Our consistent and multidisciplinary approach, which included retrograde perfusion during deployment and a 9-cm-long of FET anastomosed at zone 3, helped achieve acceptable outcomes for preventing SCI. Furthermore, FET is effective at shortening circulatory arrest time, as shown in both the overall cohort and the matched cohort.

A major source of concern following initial life-saving surgery for AAAD is aortic remodeling in the downstream aorta. TAR makes additional TEVAR easier because zone 2 landing is possible only with an extrathoracic bypass of the left subclavian artery. Otherwise, simple TEVAR is possible if the elephant trunk is installed. However, in the current study, nearly one-half of the patients in both groups who underwent reintervention in the downstream aorta required open surgery. This led us to believe that secondary TEVAR is not a viable option for reintervention after initial surgery for AAAD, and we began to consider methods to promote aortic remodeling following initial surgery.

d-SINE is not insignificant as a predictor of reintervention. Hiraoka and colleagues^20^ reported a 5-year incidence of d-SINE as high as 21%, implying a link between d-SINE and the distal angle of the stent graft. They also demonstrated that FET implantation initiates d-SINE more frequently in the subacute or chronic phase compared to the acute phase. d-SINE becomes more concerning when the stent graft is positioned in an area prone to flexion of the major curvature of the proximal descending aorta, with a shorter length of FET and proximalization of the distal anastomosis of the TAR to zone 0-2 for subacute or chronic dissection. In the current study, TAR + FET in the acute phase was performed with zone 3 anastomosis using a 9-cm FET. This strategy was justified by the lower incidence of d-SINE (3.3%; n = 3/90) along with the reduced SCI complications and maximized aortic remodeling.

In the current study, the long-term prevention of distal aortic reoperation and the aortic remodeling effect were found to be superior after TAR + FET compared to TAR + CET. We identified aortic diameter at the CA level in the early postoperative period as a risk factor for reoperation.^5^ It should be noted that the aortic diameter at the CA level was significantly smaller after TAR + FET in this study.

### Limitations

The current study was a nonrandomized single-center study, and the 2 groups had different follow-up periods owing to the study's retrospective nature. Not all patients were diagnosed using contrast-enhanced CT to evaluate serial aortic size changes and the presence of d-SINE. The relatively small number of patients and varying follow-up periods might have resulted in type II errors. Selection bias remained even after PSM analysis. Moreover, the PSM included only 8 preoperative variables, which is insufficient for full elucidation. Future studies with larger and more closely matched cohorts are needed to evaluate middle- to long-term outcomes.

## Conclusions

Compared to conventional TAR with a CET, TAR with an FET requires a shorter circulatory arrest time and in our cohort was not associated with permanent SCI or the need for distal aortic reintervention for aortic enlargement at the downstream aorta at mid-term follow-up. FET has potential as the first option for improving mid-term outcomes after surgery for AAAD.

## Conflict of Interest Statement

The authors reported no conflicts of interest. The *Journal* policy requires editors and reviewers to disclose conflicts of interest and to decline handling or reviewing manuscripts for which they may have a conflict of interest. The editors and reviewers of this article have no conflicts of interest.

## Supplementary Data

Video 1 Brief introduction of procedures including classical elephant trunk. Video available at: https://www.jtcvs.org/article/S2666-2736(25)00231-1/fulltext.
