# Supplementary figures and images for: Efficacy of total arch replacement with frozen elephant trunk in patients with acute type A aortic dissection
Source: JTCVS Open. 2025 Jul 16;27:8–16. doi: 10.1016/j.xjon.2025.07.002 (PMC12570541; doi:10.1016/j.xjon.2025.07.002)

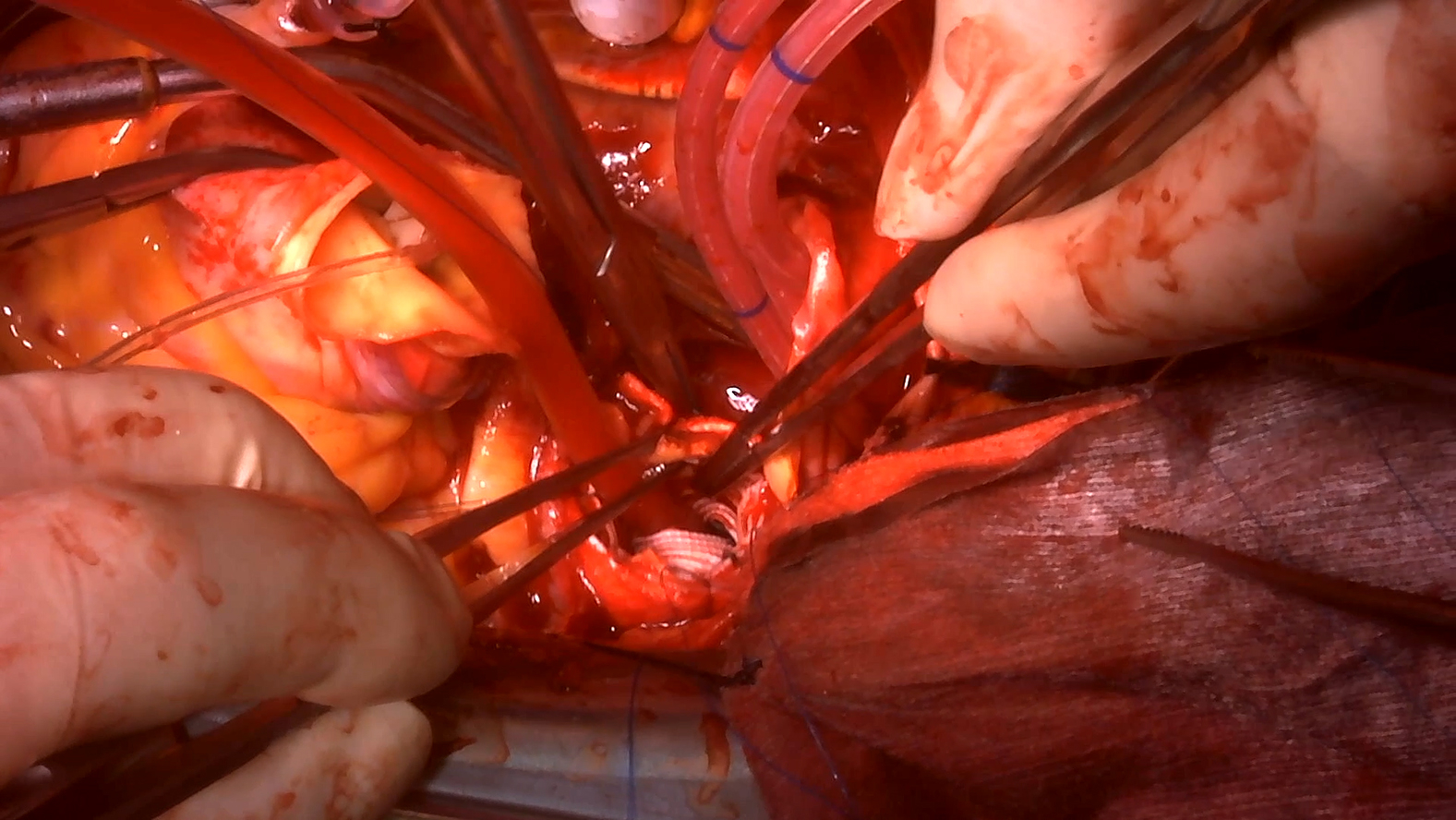

Supplement: Video 1 — Brief introduction of procedures including classical elephant trunk. Video available at: https://www.jtcvs.org/article/S2666-2736(25)00231-1/fulltext. [file fx2.jpg]
